# The effect of neoadjuvant radiotherapy on immune cell infiltrates in myxofibrosarcoma

**DOI:** 10.1016/j.iotech.2026.101586

**Published:** 2026-02-21

**Authors:** M.J.L. Nederkoorn, U.E. Flucke, M.H.S. Hillebrandt-Roeffen, M.A.J. Gorris, S.G. van Ravensteijn, K. Verrijp, P.M. Braam, J.J. Bonenkamp, E.F. Dierselhuis, E. Ruijter, C. Wetzels-van der Velden, H.V.N. Kusters-Vandevelde, C.J.R. Huysentruyt, J.M.M. Grefte, J.H.W. de Wilt, I.M.E. Desar, Y.M.H. Versleijen-Jonkers

**Affiliations:** 1 Department of Medical Oncology, Radboud University Medical Center, Nijmegen, The Netherlands; 2 Department of Pathology, Radboud University Medical Center, Nijmegen, The Netherlands; 3 Department of Medical BioSciences, Radboud University Medical Center, Nijmegen, The Netherlands; 4 Department of Medical Oncology, Catharina Hospital, Eindhoven, The Netherlands; 5 Department of Radiotherapy, Radboud University Medical Center, Nijmegen, The Netherlands; 6 Department of Surgery, Radboud University Medical Center, Nijmegen, The Netherlands; 7 Department of Orthopedic Surgery, Radboud University Medical Center, Nijmegen, The Netherlands; 8 Department of Pathology, Rijnstate Hospital, Arnhem, The Netherlands; 9 Department of Pathology, Jeroen Bosch Hospital, ‘s-Hertogenbosch, The Netherlands; 10 Department of Pathology, Canisius-Wilhelmina Hospital, Nijmegen, The Netherlands; 11 Department of Pathology, Catharina Cancer Institute—Eurofins PAMM, Eindhoven, The Netherlands; 12 Department of Pathology, Gelre Hospitals, Apeldoorn, The Netherlands

**Keywords:** myxofibrosarcoma, soft-tissue sarcoma, neoadjuvant radiotherapy, tumor immune microenvironment, immune infiltrate

## Abstract

**Background:**

Myxofibrosarcoma (MFS) is a subtype of soft-tissue sarcoma for which local treatment includes neoadjuvant radiotherapy (nRT) followed by surgery. The impact of nRT on the MFS tumor immune microenvironment remains unexplored.

**Materials and methods:**

Paired pre-nRT biopsy and post-nRT surgery samples from 31 MFS patients were retrospectively collected. The intratumoral density of T cells, cytotoxic T cells, regulatory T cells, helper T cells, B cells and natural killer (NK) cells and expression of programmed death-ligand 1 were quantified using multiplex immunohistochemistry (mIHC). mIHC marker densities were compared between pre- and post-nRT samples, and their associations with clinicopathological characteristics and patient outcomes were assessed.

**Results:**

There was substantial interpatient heterogeneity in mIHC marker densities, both pre- and post-nRT. A significant reduction in cytotoxic T cell {205 [interquartile range (IQR) 674] pre-nRT versus 58 (IQR 205) post-nRT, *P* = 0.030} and helper T cell [220 (IQR 343) pre-nRT versus 74 (IQR 110) post-nRT, *P* = 0.011] densities, alongside an increase in NK cell [2 (IQR 8) pre-nRT versus 7 (IQR 16) post-nRT, *P* = 0.050] infiltration, was observed following nRT. There were no statistically significant associations between mIHC marker densities measured before or after nRT, or their changes over time, and patient outcomes.

**Conclusions:**

Conventional nRT is associated with reduced cytotoxic and helper T cell infiltration and increased presence of NK cells in MFS. These results suggest that conventional nRT reduces inflammatory aspects of MFS tumors.

## Highlights

• Comparison of paired pre- and post-nRT MFS samples revealed nRT-induced changes in intratumoral lymphocyte densities.
• Both pre- and post-nRT MFS samples showed strong inter‑patient heterogeneity in intratumoral lymphocyte infiltration.
• Conventional nRT was associated with reduced intratumoral cytotoxic T-cell and helper T-cell densities in MFS.
• MFS samples obtained after nRT harbored increased intratumoral NK cell densities.
• An increase in intratumoral T-cell densities after nRT was associated with a trend toward improved outcomes in MFS.

## Introduction

Myxofibrosarcoma (MFS) is a soft-tissue sarcoma (STS) subtype characterized by multinodular infiltrative local growth. It represents ∼3% of total STS diagnoses with an age-standardized incidence rate of 0.19 per 100 000 person-years in Europe.^1^^,^^2^ MFS typically presents in the extremities of elderly patients as a tumor of high histological grade.^3^^,^^4^ Conventional curative treatment for local disease comprises wide surgical excision, which is frequently combined with adjuvant or neoadjuvant radiotherapy (nRT) to improve local control.^5^

Despite adequate treatment, local recurrence (LR) is reported in 16%-39% of patients.^3^^,^^4^^,^^6^^,^^7^ Distant metastases are more likely to occur in patients with tumors of high histological grade and affect 17%-38% of patients.^3^^,^^6^^,^^7^ The 5-year overall survival for MFS has been reported to range from 71% to 84%.^3^^,^^4^^,^^6^^,^^7^ The high risk for LR despite aggressive local treatment and poor outcomes in advanced or metastatic disease highlight the clinical need for improved therapeutic approaches.

Immune checkpoint inhibitors (ICIs) have revolutionized the therapeutic landscape and improved clinical outcomes for various cancers, but not yet for patients with MFS. Recent clinical evidence has highlighted the efficacy of ICIs in select subgroups of STS.^8^, ^9^, ^10^, ^11^, ^12^, ^13^, ^14^, ^15^ Responses were most frequently observed in dedifferentiated liposarcoma (ddLPS), undifferentiated pleomorphic sarcoma (UPS) and alveolar soft-part sarcoma. ICIs have also indicated promising activity in a large fraction of MFS, but their clinical effectiveness remains mostly unexplored and warrants further investigation. Despite encouraging responses reported in these subgroups, most STS patients derived no meaningful benefit from ICIs in monotherapy.

RT is capable of remodeling the tumor microenvironment by inducing tumor cell death and local inflammation. In exceedingly rare cases, this can lead to a systemic antitumor immune response, referred to as the abscopal effect.^16^ In a clinical setting, the combination of RT and ICI demonstrated superior activity and outcomes across multiple clinical trials.^17^^,^^18^ Especially relevant in this context is the SU2C-SARC032 trial, which reported a superior disease-free survival (DFS) in patients treated with pembrolizumab and nRT with consecutive surgery in UPS, ddLPS and pleomorphic liposarcoma.^19^ However, the synergistic effects of ICI and RT are not observed in all cancers, as several clinical trials investigating this combination yielded dissatisfying results.^20^^,^^21^ In line with these observations, preclinical studies reported heterogeneous changes in the immune contexture of the tumor microenvironment of several STS subtypes following RT treatment.^22^, ^23^, ^24^, ^25^ Specific STS subtypes showed an increase in tumor-infiltrating lymphocytes (TILs) following RT, whereas other subtypes revealed no consistent change. These findings suggest that the synergistic potential of RT and ICI might be STS subtype specific. The immunomodulatory effects of RT have not yet been specifically explored for MFS.

A deeper understanding of the immunomodulatory impact of RT on the MFS tumor microenvironment will provide further insights into the rationale for combining RT and immunotherapy in this specific STS subtype. We therefore carried out a quantitative evaluation of conventional nRT-induced changes in intratumoral lymphocytes and programmed death-ligand 1 (PD-L1) expression in paired pre- and post-nRT MFS tissue samples as a first step towards the potential use of RT and ICI combinations.

## Materials and methods

### Patients and tissue preparation

Patients diagnosed with MFS and treated with nRT followed by surgery within the Radboud University Medical Center between 2002 and 2023 were identified through a search in the Dutch nationwide network and registry of histo- and cytopathology database (PALGA). From these patients, paired pre-nRT diagnostic and post-nRT excision formalin-fixed paraffin-embedded (FFPE) samples were collected. Samples were histopathologically reviewed by a dedicated sarcoma pathologist (UEF) and representative pre- and post-nRT regions with adequate viable tumor cells were selected for further analysis. Samples lacking adequate viable tumor cells or containing large necrotic areas were excluded from further analyses. When deemed feasible, tissue microarrays (TMAs) were constructed using two to three 2-mm core punches to account for spatial heterogeneity. If the construction of TMAs was unfeasible, analyses were conducted using whole biopsy slides. Samples with a total tissue surface area <1 mm^2^ were considered inadequate and excluded from the analysis.

### Clinical data

All participating patients provided written informed consent for use of their clinical data and tissue samples according to institutional guidelines, with ethical approvals obtained from the local certified Medical Ethics Committee (2016-2686). Clinical data were retrieved from a prospective clinical registry (NCT05373810) and supplemented with information from PALGA. Patients were classified as having a complete pathological response to nRT based on a ≥95% tumor necrosis threshold.^26^^,^^27^

### Multiplex immunohistochemistry and data acquisition

TMAs and whole-tissue FFPE samples were sectioned at a 4-μm thickness and mounted on to SuperFrost Plus slides (VWR, Radnor, PA, USA). Multiplex immunohistochemistry (mIHC) using two panels was carried out using a Bond RX autostainer (Leica Biosystems, Nussloch, Germany).

**Figure 1:**
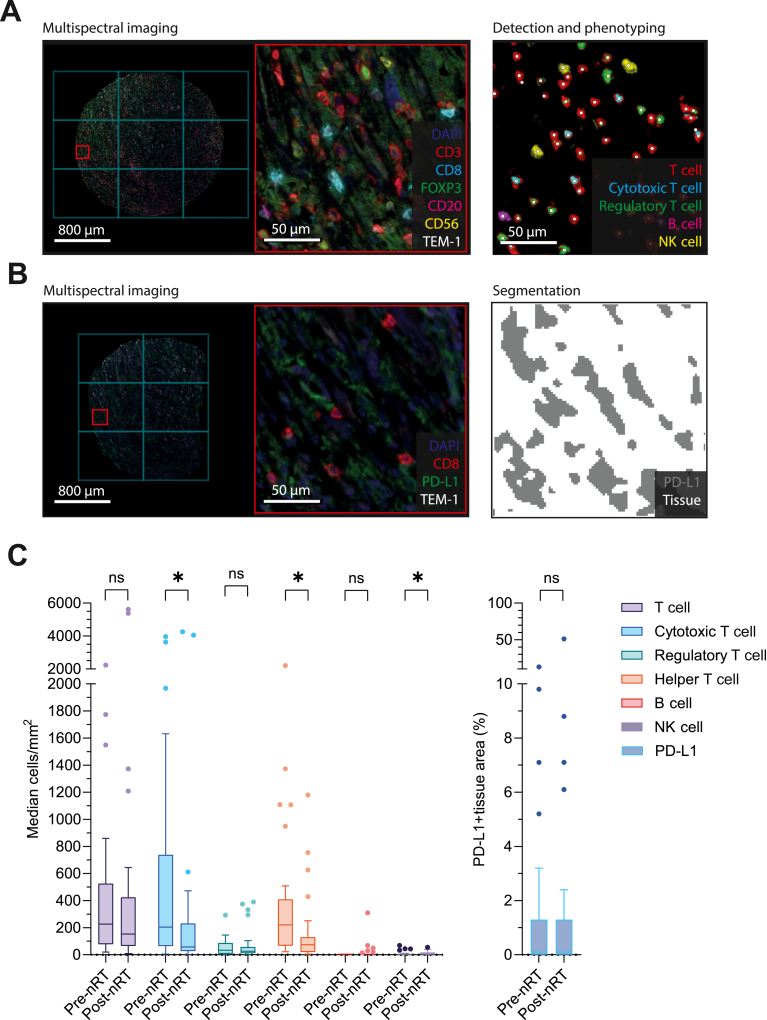
**Characterization of intratumoral lymphocyte infiltration and programmed death-ligand 1 (PD-L1) expression in paired pre- and post-neoadjuvant radiotherapy (nRT) myxofibrosarcoma tissue samples by multiplex immunohistochemistry.** (A) Multispectral imaging, detection and phenotyping of tumor-infiltrating lymphocyte subtypes. (B) Multispectral imaging and segmentation for PD-L1-expressing tissue area. (C) Absolute median tumor-infiltrating lymphocyte subtype densities and median percentage of PD-L1-expressing tissue area. Lines and error bars represent the median and interquartile range. ns, not significant. ∗, *P*≤0.05.

The first mIHC panel was used to stain different lymphocyte populations as described before (Figure 1A).^28^^,^^29^ The second mIHC panel was stained with the following sequence: anti-programmed cell death protein 1 (PD-1) (Cell Signaling Technology, Danvers, MA, USA, 86163, clone D4W2J, 1 : 100) with Opal620, anti-CD8 (Agilent Dako, Santa Clara, CA, USA, M7103, clone C8/144B, 1 : 1600) with Opal520, anti-PD-L1 (Cell Signaling, 13684, clone E1L3N, 1 : 500) with Opal570, anti-LAG-3 (Abcam, Cambridge, UK, ab180187, clone EPR4392, 1 : 5000) with Opal520, anti-TIM-3 (R&D systems, Minneapolis, MN, USA, MAB23652, clone 2321C, 1 : 50) (Figure 1B). An antibody against TEM-1 (Abcam, ab204914, clone EPR17081, 1 : 2000) was included in both mIHC panels as a tumor marker.^30^ Multispectral images were acquired using a PhenoImager HT (Akoya Biosciences, Marlborough, MA, USA) at ×20 magnification using Vectra Polaris software (V1.0.13, Akoya Biosciences). All tissue-containing images were selected for inForm batch processing using Phenochart software (V1.2.0, Akoya Biosciences). Representative images were selected using Phenochart software to train an algorithm for spectral unmixing of Opal fluorophores, removal of autofluorescence signal and tissue segmentation using inForm image analysis software (V2.6.0, Akoya Biosciences). For the segmentation of tissue from background, an algorithm was trained to recognize tumor and background based on DAPI, TEM-1 and autofluorescence channels. For the segmentation of PD-L1-expressing tissue, an algorithm was trained to recognize tumor, PD-L1-expressing tissue and background based on DAPI, PD-L1, TEM-1 and autofluorescence channels (Figure 1B). These algorithms were used to batch process all tissue-containing regions.

Cell identification for the lymphocyte panel was carried out using ImmuNet software, as described before (Figure 1A).^31^^,^^32^ Due to the technical limitations of the ImmuNet software in its current state, the exhaustion markers (PD-1, TIM-3 and LAG-3) could not be reliably analyzed for this study. Lymphocyte subsets were classified as general T cells (CD3+), cytotoxic T cells (CD3+ and CD8+), regulatory T cells (CD3+ and FOXP3+), helper T cells (CD3+, CD8− and FOXP3−), B cells (CD20−) or natural killer (NK) cells (CD56+). Lymphocyte subset densities (cells/mm^2^) were calculated by dividing absolute subset count by tissue surface area. The percentage of tissue area positive for PD-L1 staining was quantified per TMA or tissue region using inForm binary segmentation data, which was processed by ImageJ and loaded into QuPath software (V0.5.1) and analyzed through pixel classification.^33^

### Statistical analyses

Normality of continuous variables was assessed using the Shapiro–Wilk test. Data were described using median values with the interquartile range (IQR). DFS was modeled using the Kaplan–Meier method, and differences across groups were compared using the log-rank test. Paired non-normal data were compared using the two-tailed related-samples Wilcoxon signed-rank test. Differences in event occurrence across categorical groups were assessed using the chi-square test. Findings were considered statistically significant with a *P* value ≤0.05. Statistical analyses were carried out using SPSS Statistics (V29.0.0.0, IBM). Data were visualized using GraphPad Prism (V10.4.1, GraphPad Software).

## Results

### Clinicopathological characteristics

The clinicopathological characteristics of the MFS patient cohort (*N* = 31) are presented in Table 1. Median age at diagnosis was 64 (IQR 16) years and 13 (42%) patients were female. The majority of tumors were located deep (84%) and were of high histological grade (97%). The most frequently applied nRT schedule comprised 50 Gray (Gy) applied throughout 25 fractions of 2 Gy (77.4%). Median interval between the end of nRT and surgery was 57 (IQR 19) days. Six patients (19.4%) had ≥95% necrosis in response to nRT. None of the patients included in the current study received (neo)adjuvant systemic therapy. Median follow-up was 40 (IQR 29) months and one patient (3%) experienced an LR, six patients (19%) developed distant metastases and six patients (19%) died.

**Table 1:** Clinicopathological characteristics of myxofibrosarcoma patients treated with nRT and surgery

Patient characteristics	*N = 31*
Sex, *n* (%)	
Male	18 (58)
Female	13 (42)
Median age at diagnosis, years (IQR)	64 (16)
Median follow-up, months (IQR)	40 (29)
Tumor location, *n* (%)	
Upper arm	1 (3.2)
Lower arm	3 (9.7)
Upper leg	16 (51.6)
Lower leg	8 (25.8)
Pelvis	2 (6.5)
Trunk	1 (3.2)
Tumor depth, *n* (%)	
Superficial	5 (16)
Deep	26 (84)
Tumor grade, *n* (%)	
High grade	30 (97)
Grade 2	1 (3)
Median tumor size, mm (IQR)	70 (79)
Radiotherapy schedule, *n* (%)	
50 Gy (25 × 2 Gy)	24 (77.4)
45 Gy (15 × 3 Gy)	6 (19.4)
25 Gy (5 × 5 Gy)	1 (3.2)
Median interval between nRT and surgery, days (IQR)	57 (19)
Classification surgical margin, *n* (%)	
R0	26 (83.9)
R1	3 (9.7)
R2	2 (6.5)
Pathological response to nRT (necrosis), *n* (%)	
≥95%	6 (19.4)
<95%	23 (74.2)
Unknown	2 (6.5)
Local recurrence, *n* (%)	
Yes	1 (3)
No	30 (97)
Metastatic disease, *n* (%)	
Yes	6 (19)
No	25 (81)
Deceased, *n* (%)	
Yes	6 (19)
No	25 (81)

Gray, Gy; IQR, interquartile range; nRT, neoadjuvant radiotherapy.

### Characterization of intratumoral lymphocyte densities and PD-L1 expression

The intratumoral presence of TIL and expression of PD-L1 were characterized using mIHC (Figure 1, and Tables 2 and 3). There was substantial interpatient heterogeneity in TIL densities and PD-L1 expression (Figure 1C). In general, T cell subsets were detected at higher densities compared with B cells or NK cells. PD-L1 was detected in at least 1% of the total analyzed area in 8 patients (26%) pre-nRT and 10 patients (32%) post-nRT.

**Table 2:** Median absolute densities of tumor-infiltrating lymphocyte subtypes in paired pre- and post-nRT myxofibrosarcoma tissue samples

Lymphocyte subset	Pre-nRT density, cells/mm^2^ (IQR)	Post-nRT density, cells/mm^2^ (IQR)	*P* value	Median Δ-marker density (IQR)
T cell	227 (449)	153 (360)	0.938	1.0 (2.6)
Cytotoxic T cell	205 (674)	58 (205)	0.030^a^	0.4 (1.4)
Regulatory T cell	34 (80)	26 (47)	0.791	0.7 (2.5)
Helper T cell	220 (343)	74 (110)	0.011^a^	0.4 (1.1)
B cell	2 (1)	0 (3)	0.441	0.0 (1.5)
NK cell	2 (8)	7 (16)	0.050^a^	2.5 (8.2)

Δ-marker density, change in multiplex immunohistochemistry marker density; IQR, interquartile range; NK, natural killer; nRT, neoadjuvant radiotherapy.

a *P*≤0.05.

**Table 3:** Median PD-L1-expressing tissue area in paired pre- and post-nRT myxofibrosarcoma tissue samples

Marker	Pre-nRT positive surface area,% (IQR)	Post-nRT positive surface area, % (IQR)	*P* value	Median Δ-marker density (IQR)
PD-L1-expressing tissue area	0.1 (1.3)	0.1 (1.3)	0.657	1.0 (2.0)

Δ-marker density, change in multiplex immunohistochemistry marker density; IQR, interquartile range; nRT, neoadjuvant radiotherapy; PD-L1, programmed death-ligand 1.

Lymphocyte subsets and PD-L1 expression were compared across clinicopathological subgroups to identify associations between TIL densities or the expression of PD-L1 and specific clinicopathological features. Significant associations between mIHC marker densities and clinicopathological features were limited to a difference in pre-nRT helper T cell density between patients with deep and superficial tumors. Patients with deep tumors (*n* = 26, 84%) harbored a significantly higher median pre-nRT helper T cell density compared with patients with superficial tumors (*n* = 5, 16%) [285 (IQR 417) versus 55 (IQR 184), *P* = 0.031]. This difference in median helper T cell density was not observed post-nRT [70 (IQR 98) versus 75 (IQR 686), *P* = 0.696].

Patients were stratified into mIHC marker high or low subgroups using the respective median marker density as a cut-off to evaluate their association with DFS. Associations between pre- or post-nRT mIHC marker density subgroups and DFS did not reach statistical significance (Supplementary Figure S1, available at https://doi.org/10.1016/j.iotech.2026.101586).

### Radiotherapy induces changes in the intratumoral immune landscape

**Figure 2:**
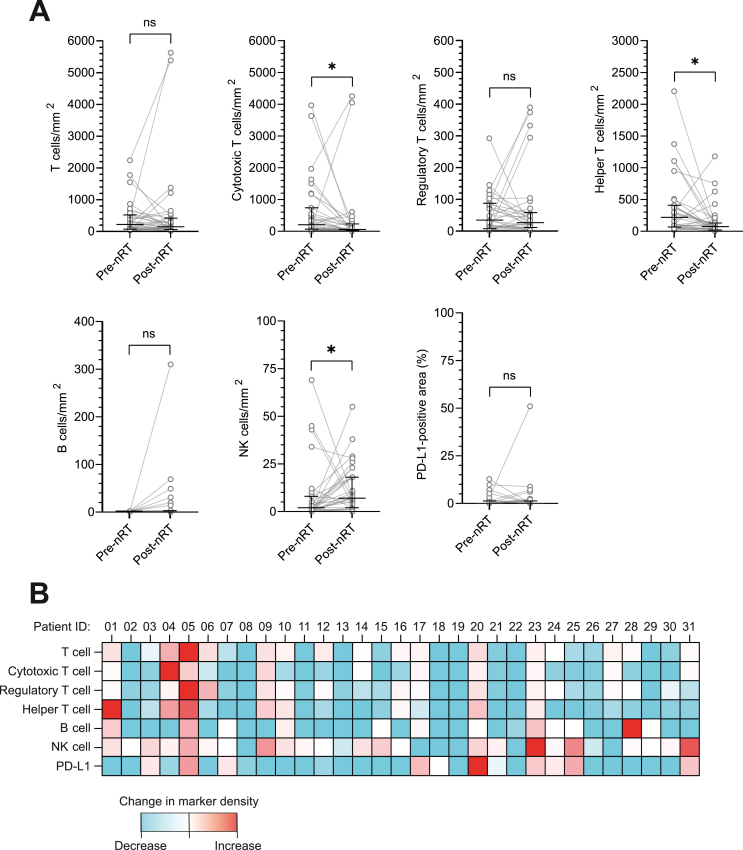
**Changes in intratumoral lymphocyte infiltration and programmed death-ligand 1 (PD-L1) expression between paired pre- and post-neoadjuvant radiotherapy (nRT) myxofibrosarcoma tissue samples.** (A) Changes in the absolute median density of T cells, cytotoxic T cells, regulatory T cells, helper T cells, B cells and NK cells, and the change in median PD-L1-positive tissue area following nRT. Lines and error bars represent the median and interquartile range. (B) Changes in tumor-infiltrating lymphocyte subsets and PD-L1-expressing area following nRT per patient. ns, not significant. ∗, *P*≤0.05.

The effects of conventional nRT on the intratumoral presence of TIL and expression of PD-L1 were characterized by comparing mIHC marker densities between paired pre-nRT and post-nRT samples (Tables 2 and 3, and Figure 2). Post-nRT samples comprised significantly lower median densities of cytotoxic T cells [205 (IQR 674) pre-nRT versus 58 (IQR 205) post-nRT cells/mm^2^, *P* = 0.030] and helper T cells [220 (IQR 343) pre-nRT versus 74 (IQR 110) post-nRT cells/mm^2^, *P* = 0.011] compared with paired pre-nRT samples. Furthermore, post-nRT samples harbored significantly higher densities of NK cells [2 (IQR 8) pre-nRT versus 7 (IQR 16) post-nRT cells/mm^2^, *P* = 0.050] compared with corresponding pre-nRT samples. The median change in marker density (Δ-marker density) was negative for general T cells, cytotoxic T cells, regulatory T cells, helper T cells and B cells. In contrast, NK cells revealed a positive Δ-marker density (Tables 2 and 3, and Figure 2). To assess whether these changes in mIHC marker densities are dependent on nRT response, changes in marker densities were compared for patients with <95% and ≥95% pathological nRT response. Interestingly, patients with a <95% pathological nRT response (*n* = 23) showed a significant reduction in cytotoxic T cell, helper T cell and B cell densities after nRT, whereas this difference was not detected in patients with a ≥95% pathological response (*n* = 6) (Supplementary Table S1, available at https://doi.org/10.1016/j.iotech.2026.101586).

The change in mIHC marker densities was compared across clinicopathological subgroups to identify relations between clinicopathological characteristics and a directed change in TIL densities or the expression of PD-L1. Patients with a ≥95% pathological nRT response had a significantly larger reduction in PD-L1 expression compared with patients with a <95% pathological response [0.00 (IQR 0.13), *n* = 6 versus 0.50 (IQR 9.50), *n* = 25]. No other associations between the change in mIHC marker densities and clinicopathological subgroups were detected.

To further investigate the association between the Δ-marker density and patient outcomes, patients were stratified into mIHC marker density increase or decrease groups, using a twofold positive or negative change as the respective cut-off values.

There were no statistically significant associations between DFS and a twofold change in mIHC marker densities (Supplementary Figure S2, available at https://doi.org/10.1016/j.iotech.2026.101586). However, a trend towards an improved DFS is observed in patients with a twofold increase in general T cells, cytotoxic T cells, regulatory T cells, helper T cells and B cells. This trend was associated with a lower incidence of distant metastases (Supplementary Table S2, available at https://doi.org/10.1016/j.iotech.2026.101586).

## Discussion

Comparison of paired pre- and post-nRT tissue samples revealed that conventional nRT is associated with a decrease in intratumoral cytotoxic T cell and helper T cell densities in MFS. Conversely, there was a minor increase in NK cell densities following nRT. In the current cohort of MFS patients, intratumoral pre- or post-nRT lymphocyte densities or expression of PD-L1, or the change in these respective markers following conventional nRT, were not associated with patient outcomes.

Previous research on the immunomodulatory effects of RT primarily focused on heterogeneous groups of STS entities or smaller groups of specific subtypes. van Oost et al. investigated the effects of nRT in a cohort consisting of paired pre- and post-nRT samples from 13 UPS and 8 MFS patients.^25^ In contrast to the findings presented in the current study, the authors reported that conventional nRT did not induce significant changes in the MFS tumor immune microenvironment. These aberrant findings might be explained by the smaller MFS cohort size, which might not fully reflect the large immunologic interpatient heterogeneity. The authors emphasize important differences in the immune landscape between MFS and UPS. In contrast to the absence of change observed in MFS, they reported an increase in cytotoxic T cell and myeloid cell infiltration following nRT in UPS. This observation is consistent with the findings of Keung et al., who reported that nRT is associated with a trend towards an increase in T cell, cytotoxic T cell, helper T cell and regulatory T cell densities in UPS.^22^ These findings suggest important differences in immune contexture and immune-related response to RT between UPS and MFS, despite their (epi)genetic and morphologic similarities.^34^^,^^35^ The substantial overlap between high-grade MFS and UPS makes it challenging to discern these entities. Therefore, studies focused on either entity, including the present research, should consider the possibility that the cohort might comprise cases of the alternative identity. Another study examined the immunomodulatory effects of nRT, with or without chemotherapy, in a cohort of 32 STS, including a high proportion of UPS cases.^23^ They observed a general increase in tumor-infiltrating immune cells and a relative increase in monocytes, macrophages, B cells and helper T cells. Furthermore, Rupp et al. reported that post-treatment samples generally comprised lower densities of T cells and cytotoxic T cells in a heterogeneous cohort of 20 STS treated with nRT, locoregional hyperthermia and occasionally chemotherapy. This observation could not be confirmed in a paired comparison of pre- and post-treatment samples.^24^

Patients were categorized into groups with a good or poor nRT response using a ≥95% necrosis threshold. This cut-off value has been applied throughout multiple studies in STS to define a favorable pathological response to nRT but remains controversial as its prognostic significance has not been established.^26^^,^^27^ In the current study, pre-nRT lymphocyte densities or PD-L1 expression were not predictive of a ≥95% pathological nRT response. In line with the complete cohort, patients with a <95% pathological response revealed a significant decrease in intratumoral cytotoxic T cell, helper T cell and B cell densities. Notably, these changes in TIL densities were not observed in the six patients with a ≥95% pathological response to nRT. These findings indicate that the reduction in cytotoxic T cells and helper T cells observed in the complete cohort is driven by patients with a <95% pathological response to nRT. Despite the limited number of patients, this might indicate that immune preservation is an indicator for a ≥95% pathological response following nRT. Previous studies in other STS subtypes reported no associations between changes in TIL densities and response to nRT.^25^ However, one study found a significant increase in monocyte infiltration in STS samples with >90% necrosis following nRT, with or without administering chemotherapy.^23^ Overall, these studies reflect the immunologic heterogeneity across STS subtypes and highlight that this heterogeneity is also reflected by the variable immunologic responses of these histotypes to RT. These findings emphasize the need for histotype-tailored research to guide future immunotherapy trials, especially in combination with RT.

Recent clinical trials have provided further insights into the rationale for the combination of RT and immunotherapy in STS. Mowery et al. compared the effects of neoadjuvant pembrolizumab with concurrent RT followed by surgery with nRT and surgery alone in a randomized phase II study comprising 143 patients with extremity STS.^19^ The authors reported a superior 2-year DFS of 67% for the experimental cohort (*n* = 64), compared with 52% for the control group (*n* = 63). The experimental cohort comprised seven patients with MFS, who were included in the broader category of UPS. For this UPS/MFS cohort, although statistically underpowered, the DFS of the experimental group was superior to the control group (hazard ratio 0.67, *P* = 0.17). Furthermore, Levy et al. reported a phase II clinical study in which they investigated anti-PD-L1 antibody atezolizumab with concurrent stereotactic body RT in a cohort of 61 STS patients, of whom 60 were pretreated with chemotherapy. The median progression-free survival and overall survival were 2.5 and 8.6 months, respectively. They reported that infiltration by tumor-associated macrophages and monocyte-derived cells following RT was negatively associated with clinical outcomes. There was no difference in lymphocyte population densities between responders and non-responders.^36^ Another study by Roland et al. investigated neoadjuvant nivolumab or ipilimumab and nivolumab in 27 patients with ddLPS and UPS of the extremity and trunk in a randomized non-comparative phase II clinical trial.^14^ Patients with UPS received concurrent nRT, whereas patients with ddLPS did not receive nRT. The UPS cohort revealed a superior pathological response compared with a historical reference population treated with conventional nRT. This effect was less pronounced for the ddLPS group. They also reported that non-responders had higher regulatory T cells at baseline compared with responders. Furthermore, the authors report differences in immune contexture between UPS and ddLPS. These clinical studies highlight the potential of combined RT and immunotherapy but also emphasize the heterogeneous responses and immunological patterns across STS subtypes. Therefore, future prospective studies should focus on specific histological subtypes to provide further insights to optimize this combination. The findings in the present research suggest that conventional nRT generally reduces the inflammatory environment of MFS. Although the immunologic context of STS is highly complex, these findings provide less rationale to combine nRT and ICI in MFS compared with other STS subtypes.

The current research focused on characterizing changes in intratumoral lymphocyte infiltration and expression of PD-L1. Although these features reflect relevant aspects of antitumor immunity, the overall immune response and its modulation by nRT are affected by a multitude of factors. Future studies should also incorporate investigations of the peritumoral compartment and additional markers for antitumor immunity, including assessment of tertiary lymphoid structures (TLS). The presence of TLS has been described as one of the strongest predictors for immunotherapy response in STS.^13^^,^^37^ However, recent clinical trials investigating the combination of immunotherapy and RT in STS have demonstrated conflicting results on the correlation of the presence of TLS and outcomes.^14^^,^^36^ The detection of these spatial structures is challenging due to their relatively low abundance and is therefore subjected to high rates of false negatives. Due to its retrospective design and limited size of the tissue samples, the detection of TLS was not feasible throughout the current study. Other relevant modulators of antitumor immunity include the intratumoral infiltration of monocytes and dendritic cells (DCs). Clinical evidence has highlighted the prominent role of monocytes in response to immunotherapy.^11^^,^^36^^,^^38^ While we observed a significant reduction in intratumoral cytotoxic T cells and helper T cells following nRT, there was a small increase in the infiltration of NK cells. This dissociation suggests that NK cell infiltration is independent from the infiltration of T cell subsets. DCs play a key role in orchestrating the adaptive antitumor immune response by priming cytotoxic T cells. Evidence suggests that crosstalk between DC and NK cells through FLT3 is associated with enhanced immune cell infiltration, survival and susceptibility to immunotherapy and its combination with RT.^39^, ^40^, ^41^ The current cohort showed no clear evidence for this crosstalk since there was no association between the infiltration of NK cells and T cell subsets. The presence of DCs in MFS has been reported, but their role within the MFS tumor microenvironment remains largely unexplored.^42^ Further research into the intratumoral presence of DCs in MFS and their change in response to nRT will provide insights into the engagement of adaptive antitumor immunity. Especially relevant in the context of immunotherapy-based treatments are markers for immune exhaustion, which provide information on the functional state of the intratumoral immune infiltrate. Evidence shows that exhaustion markers PD-1, LAG-3 and TIM-3 are widely expressed in the MFS tumor microenvironment, suggesting an immune exhausted phenotype.^25^^,^^34^^,^^43^^,^^44^ The effects of nRT on the expression of these markers for immune exhaustion in MFS remain unexplored. Further insights into the effects of nRT on the expression of these markers will clarify the rationale and mechanisms for the combination of RT and ICI in MFS. *In vivo* imaging strategies might address several limitations associated with inherently invasive tissue-based evaluation of the tumor immune microenvironment. Such strategies could facilitate non-invasive longitudinal monitoring of the tumor immune microenvironment without constraints in tissue sample size and availability. The retrospective nature of the current study resulted in a cohort with a relatively low incidence of LR (3%) compared with the 16%-39% reported in literature.^3^^,^^4^^,^^6^^,^^7^ Despite being among the largest STS subtype-specific paired cohorts, this study might have insufficient statistical power to reliably detect an association between the immune contexture and clinical outcomes. Therefore, observations regarding the trend of an improved DFS in patients with an increase in specific intratumoral lymphocyte subtypes following nRT should be verified in a cohort with increased statistical power.

Lymphocytes exhibit variable degrees of radiosensitivity, with NK cells being relatively more radioresistant than T cells and B cells.^45^ This is consistent with the marginal increase in NK cell infiltration following nRT. The increase in intratumoral lymphocyte densities following RT in specific STS subtypes, such as UPS, suggests the persistence and re-infiltration of lymphocytes after irradiation. In contrast, the tumor microenvironment of MFS appears to impair lymphocyte persistence and re-infiltration after nRT. The mechanisms underlying the difference in lymphocyte retention and re-infiltration following irradiation between MFS and other STS following RT warrant further evaluation. Due to its retrospective nature, the present research was limited by the applied RT schedules. Previous research has demonstrated that RT dose, fractioning and timing significantly affect its immunomodulatory effects.^46^ Therefore, the findings reported in the present research should be interpreted within the context of the applied RT regimens. Future research should assess the immunomodulatory effects of different RT schedules to identify the optimum strategy for combination approaches with immunotherapy.

In conclusion, the current research highlights the immunomodulatory effects of conventional nRT in MFS. The findings demonstrate that conventional nRT reduces the infiltration of cytotoxic T cells and helper T cells while increasing the density of NK cells in the MFS tumor microenvironment. Although the functional state of the post-nRT intratumoral lymphocytes remains unexplored, these findings do not support the combination of ICI and nRT in MFS. Further evaluation of immune exhaustion markers and their changes in response to nRT will clarify the functional state of the immune infiltrate after nRT. The current findings should be considered for the design of prospective studies evaluating the combination of RT and immunotherapy in patients with MFS.

## References

[1] Müller J.A., Delank K.S., Laudner K. (2023). Clinical characteristics of sarcoma patients: a population-based data analysis from a German clinical cancer registry. J Cancer Res Clin Oncol.

[2] Amadeo B., Penel N., Coindre J.M. (2020). Incidence and time trends of sarcoma (2000-2013): results from the French network of cancer registries (FRANCIM). BMC Cancer.

[3] van der Horst C.A.J., Bongers S.L.M., Versleijen-Jonkers Y.M.H. (2022). Overall survival of patients with myxofibrosarcomas: an epidemiological study. Cancers.

[4] Kamio S., Matsumoto M., Nakamura M., Kawai A., Kikuta K. (2023). Epidemiologic survey of myxofibrosarcoma using data from the bone and soft tissue tumor registry in Japan. Ann Surg Oncol.

[5] Gronchi A., Miah A.B., Dei Tos A.P. (2021). Soft tissue and visceral sarcomas: ESMO–EURACAN–GENTURIS Clinical Practice Guidelines for diagnosis, treatment and follow-up. Ann Oncol.

[6] Mühlhofer H.M.L., Lenze U., Gersing A. (2019). Prognostic factors and outcomes for patients with myxofibrosarcoma: a 13-year retrospective evaluation. Anticancer Res.

[7] Pogkas A., Reichardt P., Tunn P.U., Niethard M., Werner M., Ghani S. (2024). Localized myxofibrosarcoma: a retrospective analysis of primary therapy and prognostic factors in 134 patients in a single institution. Oncologist.

[8] Tawbi H.A., Burgess M., Bolejack V. (2017). Pembrolizumab in advanced soft-tissue sarcoma and bone sarcoma (SARC028): a multicentre, two-cohort, single-arm, open-label, phase 2 trial. Lancet Oncol.

[9] Burgess M.A., Bolejack V., Schuetze S. (2019). Clinical activity of pembrolizumab (P) in undifferentiated pleomorphic sarcoma (UPS) and dedifferentiated/pleomorphic liposarcoma (LPS): final results of SARC028 expansion cohorts. J Clin Oncol.

[10] Somaiah N., Conley A.P., Parra E.R. (2022). Durvalumab plus tremelimumab in advanced or metastatic soft tissue and bone sarcomas: a single-centre phase 2 trial. Lancet Oncol.

[11] Toulmonde M., Penel N., Adam J. (2018). Use of PD-1 targeting, macrophage infiltration, and IDO pathway activation in sarcomas: a phase 2 clinical trial. JAMA Oncol.

[12] D’Angelo S.P., Mahoney M.R., Van Tine B.A. (2018). Nivolumab with or without ipilimumab treatment for metastatic sarcoma (Alliance A091401): two open-label, non-comparative, randomised, phase 2 trials. Lancet Oncol.

[13] Italiano A., Bessede A., Pulido M. (2022). Pembrolizumab in soft-tissue sarcomas with tertiary lymphoid structures: a phase 2 PEMBROSARC trial cohort. Nat Med.

[14] Roland C.L., Nassif Haddad E.F., Keung E.Z. (2024). A randomized, non-comparative phase 2 study of neoadjuvant immune-checkpoint blockade in retroperitoneal dedifferentiated liposarcoma and extremity/truncal undifferentiated pleomorphic sarcoma. Nat Cancer.

[15] van Ravensteijn S.G., de Haan J.J., Gelderblom H. (2025). Cemiplimab in locally advanced or metastatic secondary angiosarcomas (CEMangio): a phase II clinical trial and biomarker analyses. Clin Cancer Res.

[16] Orton A., Wright J., Buchmann L. (2016). A case of complete abscopal response in high-grade pleiomorphic sarcoma treated with radiotherapy alone. Cureus.

[17] Theelen W.S.M.E., Chen D., Verma V. (2021). Pembrolizumab with or without radiotherapy for metastatic non-small-cell lung cancer: a pooled analysis of two randomised trials. Lancet Respir Med.

[18] Antonia S.J., Villegas A., Daniel D. (2017). Durvalumab after chemoradiotherapy in stage III non–small-cell lung cancer. N Engl J Med.

[19] Mowery Y.M., Ballman K.V., Hong A.M. (2024). Safety and efficacy of pembrolizumab, radiation therapy, and surgery versus radiation therapy and surgery for stage III soft tissue sarcoma of the extremity (SU2C-SARC032): an open-label, randomised clinical trial. Lancet.

[20] Spaas M., Sundahl N., Kruse V. (2023). Checkpoint inhibitors in combination with stereotactic body radiotherapy in patients with advanced solid tumors: the CHEERS phase 2 randomized clinical trial. JAMA Oncol.

[21] Monk B.J., Toita T., Wu X. (2023). Durvalumab versus placebo with chemoradiotherapy for locally advanced cervical cancer (CALLA): a randomised, double-blind, phase 3 trial. Lancet Oncol.

[22] Keung E.Z., Tsai J.W., Ali A.M. (2017). Analysis of the immune infiltrate in undifferentiated pleomorphic sarcoma of the extremity and trunk in response to radiotherapy: rationale for combination neoadjuvant immune checkpoint inhibition and radiotherapy. Oncoimmunology.

[23] Goff P.H., Riolobos L., LaFleur B.J. (2022). Neoadjuvant therapy induces a potent immune response to sarcoma, dominated by myeloid and B cells. Clin Cancer Res.

[24] Rupp L., Resag A., Potkrajcic V. (2023). Prognostic impact of the post-treatment T cell composition and spatial organization in soft tissue sarcoma patients treated with neoadjuvant hyperthermic radio(chemo)therapy. Front Immunol.

[25] van Oost S., Meijer D.M., Erdem Z.B. (2025). Divergent therapeutic and prognostic impacts of immunogenic features in undifferentiated pleomorphic sarcoma and myxofibrosarcoma. Cancer Immunol Immunother.

[26] Wardelmann E., Haas R.L., Bovée J.V. (2016). Evaluation of response after neoadjuvant treatment in soft tissue sarcomas; the European Organization for Research and Treatment of Cancer–Soft Tissue and Bone Sarcoma Group (EORTC–STBSG) recommendations for pathological examination and reporting. Eur J Cancer.

[27] Bonvalot S., Wunder J., Gronchi A. (2021). Complete pathological response to neoadjuvant treatment is associated with better survival outcomes in patients with soft tissue sarcoma: results of a retrospective multicenter study. Eur J Surg Oncol.

[28] van Ravensteijn S.G., Versleijen-Jonkers Y.M.H., Hillebrandt-Roeffen M.H.S. (2022). Immunological and genomic analysis reveals clinically relevant distinctions between angiosarcoma subgroups. Cancers (Basel).

[29] Gorris M.A.J., Martynova E., Sweep M.W.D. (2023). Multiplex immunohistochemical analysis of the spatial immune cell landscape of the tumor microenvironment. J Vis Exp.

[30] de Gooyer J.M., Versleijen-Jonkers Y.M.H., Hillebrandt-Roeffen M.H.S. (2020). Immunohistochemical selection of biomarkers for tumor-targeted image-guided surgery of myxofibrosarcoma. Sci Rep.

[31] Sultan S., Gorris M.A.J., Martynova E. (2024). ImmuNet: a segmentation-free machine learning pipeline for immune landscape phenotyping in tumors by multiplex imaging. Biol Methods Protoc.

[32] Gorris M.A.J., van der Woude L.L., Kroeze L.I. (2022). Paired primary and metastatic lesions of patients with ipilimumab-treated melanoma: high variation in lymphocyte infiltration and HLA-ABC expression whereas tumor mutational load is similar and correlates with clinical outcome. J Immunother Cancer.

[33] Bankhead P., Loughrey M.B., Fernández J.A. (2017). QuPath: open source software for digital pathology image analysis. Sci Rep.

[34] Cancer Genome Atlas Research Network (2017). Comprehensive and integrated genomic characterization of adult soft tissue sarcomas. Cell.

[35] Koelsche C., Schrimpf D., Stichel D. (2021). Sarcoma classification by DNA methylation profiling. Nat Commun.

[36] Levy A., Morel D., Texier M. (2025). Monocyte-lineage tumor infiltration predicts immunoradiotherapy response in advanced pretreated soft-tissue sarcoma: phase 2 trial results. Signal Transduct Target Ther.

[37] Petitprez F., de Reyniès A., Keung E.Z. (2020). B cells are associated with survival and immunotherapy response in sarcoma. Nature.

[38] Keung E.Z., Burgess M., Salazar R. (2020). Correlative analyses of the SARC028 trial reveal an association between sarcoma-associated immune infiltrate and response to pembrolizumab. Clin Cancer Res.

[39] Kuncman Ł., Orzechowska M., Milecki T., Kucharz J., Fijuth J. (2024). High FLT3 expression increases immune-cell infiltration in the tumor microenvironment and correlates with prolonged disease-free survival in patients with non-small cell lung cancer. Mol Oncol.

[40] Bickett T.E., Knitz M., Darragh L.B. (2021). FLT3L release by natural killer cells enhances response to radioimmunotherapy in preclinical models of HNSCC. Clin Cancer Res.

[41] Régnier P., Vetillard M., Bansard A. (2023). FLT3L-dependent dendritic cells control tumor immunity by modulating Treg and NK cell homeostasis. Cell Rep Med.

[42] Soilleux E.J., Rous B., Love K. (2003). Myxofibrosarcomas contain large numbers of infiltrating immature dendritic cells. Am J Clin Pathol.

[43] Dancsok A.R., Setsu N., Gao D. (2019). Expression of lymphocyte immunoregulatory biomarkers in bone and soft-tissue sarcomas. Mod Pathol.

[44] Klaver Y., Rijnders M., Oostvogels A. (2020). Differential quantities of immune checkpoint-expressing CD8 T cells in soft tissue sarcoma subtypes. J Immunother Cancer.

[45] Paganetti H. (2023). A review on lymphocyte radiosensitivity and its impact on radiotherapy. Front Oncol.

[46] Karapetyan L., Iheagwara U.K., Olson A.C. (2023). Radiation dose, schedule, and novel systemic targets for radio-immunotherapy combinations. J Natl Cancer Inst.

