# Supplementary material for: The effect of neoadjuvant radiotherapy on immune cell infiltrates in myxofibrosarcoma
Source: Immunooncol Technol. 2026 Feb 21;30:101586. doi: 10.1016/j.iotech.2026.101586 (PMC13000487; doi:10.1016/j.iotech.2026.101586)
Supplement: Supplementary Figures and Tables [file mmc1.docx]

The effect of neoadjuvant radiotherapy on immune cell infiltrates in myxofibrosarcoma

# **Supplementary information**

**Supplementary table 1: Absolute median tumor-infiltrating lymphocyte densities in paired pre- and post-neoadjuvant radiotherapy (nRT) myxofibrosarcoma tissue samples for patients with and without a ≥95% pathologic response to neoadjuvant radiotherapy.**

| **Phenotype** | **Pre-nRT density (cells /mm2 (IQR))** | | **post-nRT density (cells /mm2 (IQR))** | | ***P*-value** | | **Median Δ-marker density (IQR)** | |
| --- | --- | --- | --- | --- | --- | --- | --- | --- |
| **Pathologic response** | **<95%**  **(n=23)** | ***≥95%***  ***(n=6)*** | **<95%**  **(n=23)** | ***≥95%***  ***(n=6)*** | **<95%**  **(n=23)** | ***≥95%***  ***(n=6)*** | **<95%**  **(n=23)** | ***≥95%***  ***(n=6)*** |
| T-cell | 282 (449) | 106 (301) | 153 (364) | 130 (254) | 0.465 | 0.917 | 0,7 (2,6) | 2,5 (5,10) |
| Cytotoxic T-cell | 205 (674) | 86  (605) | 39  (230) | 53  (55) | 0.042* | 0.249 | 0,3  (1,3) | 0,7  (1,6) |
| Regulatory T-cell | 28 (80) | 34  (51) | 23  (37) | 38  (67) | 0.330 | 0.600 | 0,6  (3,9) | 2,0  (9,0) |
| Helper T-cell | 279 (324) | 63  (130) | 74  (90) | 55  (157) | 0.021* | 0.753 | 0,3  (1,1) | 0,6  (3,2) |
| B-cell | 2  (1) | 1  (1) | 0  (2) | 1  (23) | 0.031* | 0.684 | 0,0  (1,0) | 0,5  (15,3) |
| NK cell | 2  (7) | 1  (5) | 10  (19) | 7  (13) | 0.070 | 0.043* | 2,5  (10,7) | 2,8  (6,3) |

**Supplementary table 2: Association between the change in tumor-infiltrating lymphocyte densities and incidence of metastases.** Number of patients per condition that experienced metastases.

|  | **2-fold decrease** | **no change** | **2-fold increase** | ***P*-value (χ**^2^**)** |
| --- | --- | --- | --- | --- |
| T-cell | 4/11 (36%) | 1/8 (13%) | 1/12 (8%) | 0.201 |
| Cytotoxic T-cell | 4/17 (24%) | 2/8 (25%) | 0/6 (0%) | 0.408 |
| Regulatory T-cell | 3/11 (27%) | 2/10 (20%) | 1/10 (10%) | 0.605 |
| Helper T-cell | 5/18 (28%) | 1/8 (13%) | 0/5 (0%) | 0.323 |
| B-cell | 6/19 (32%) | 0/6 (0%) | 0/6 (0%) | 0.095 |
| NK cell | 2/6 (33%) | 0/7 (0%) | 4/14 (29%) | 0.283 |
| PD-L1 expression | 3/19 (16%) | 2/3 (67%) | 1/9 (11%) | 0.089 |

**Supplementary figure 1: Associations between multiplex immunohistochemistry (mIHC) marker densities, measured before or after neoadjuvant radiotherapy (nRT), and disease-free survival (DFS).** DFS was compared between groups using the log-rank test.
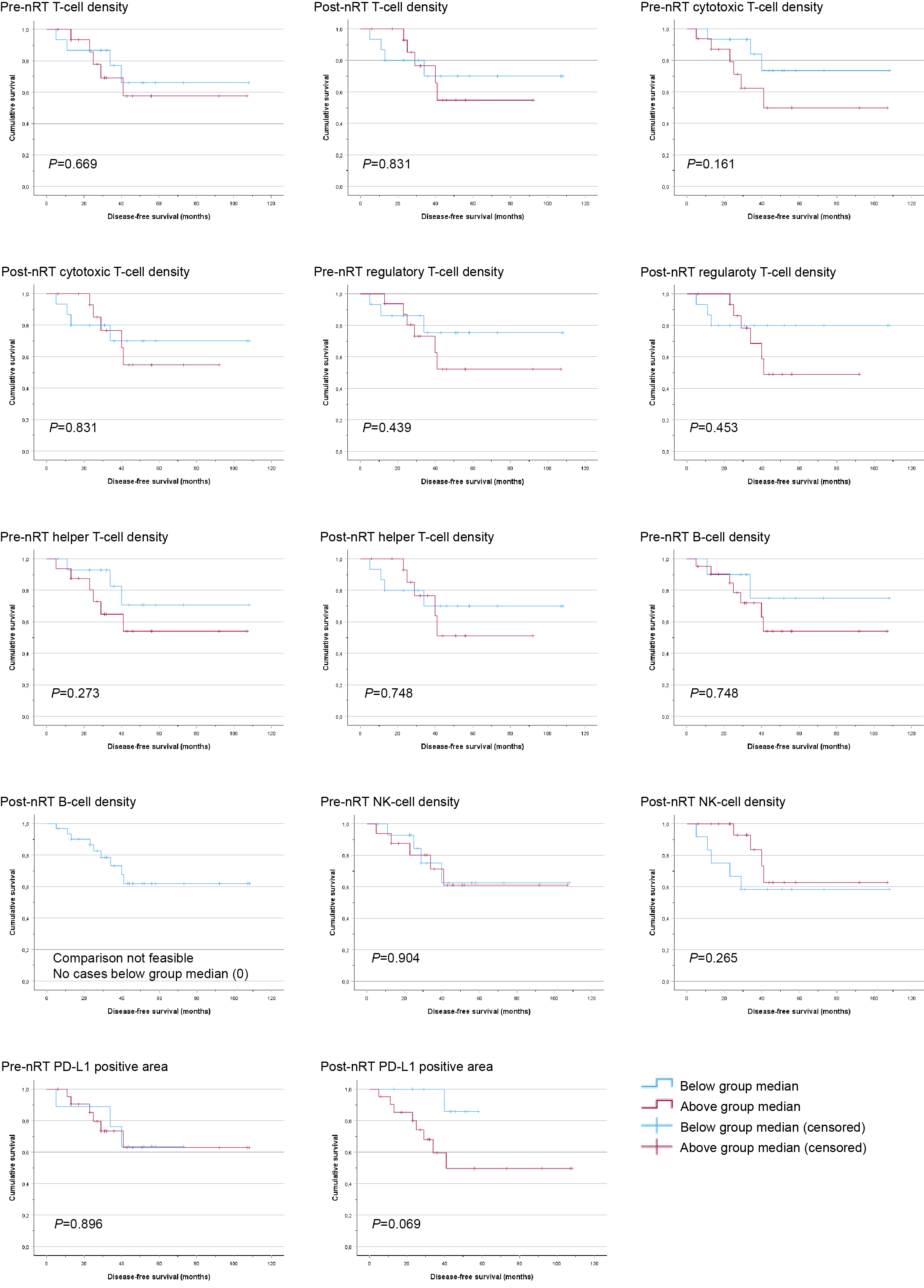


**Supplementary figure 2: Associations between the change in multiplex immunohistochemistry (mIHC) marker densities following neoadjuvant radiotherapy (nRT) and disease-free survival (DFS).** DFS was compared between groups using the log-rank test.

***
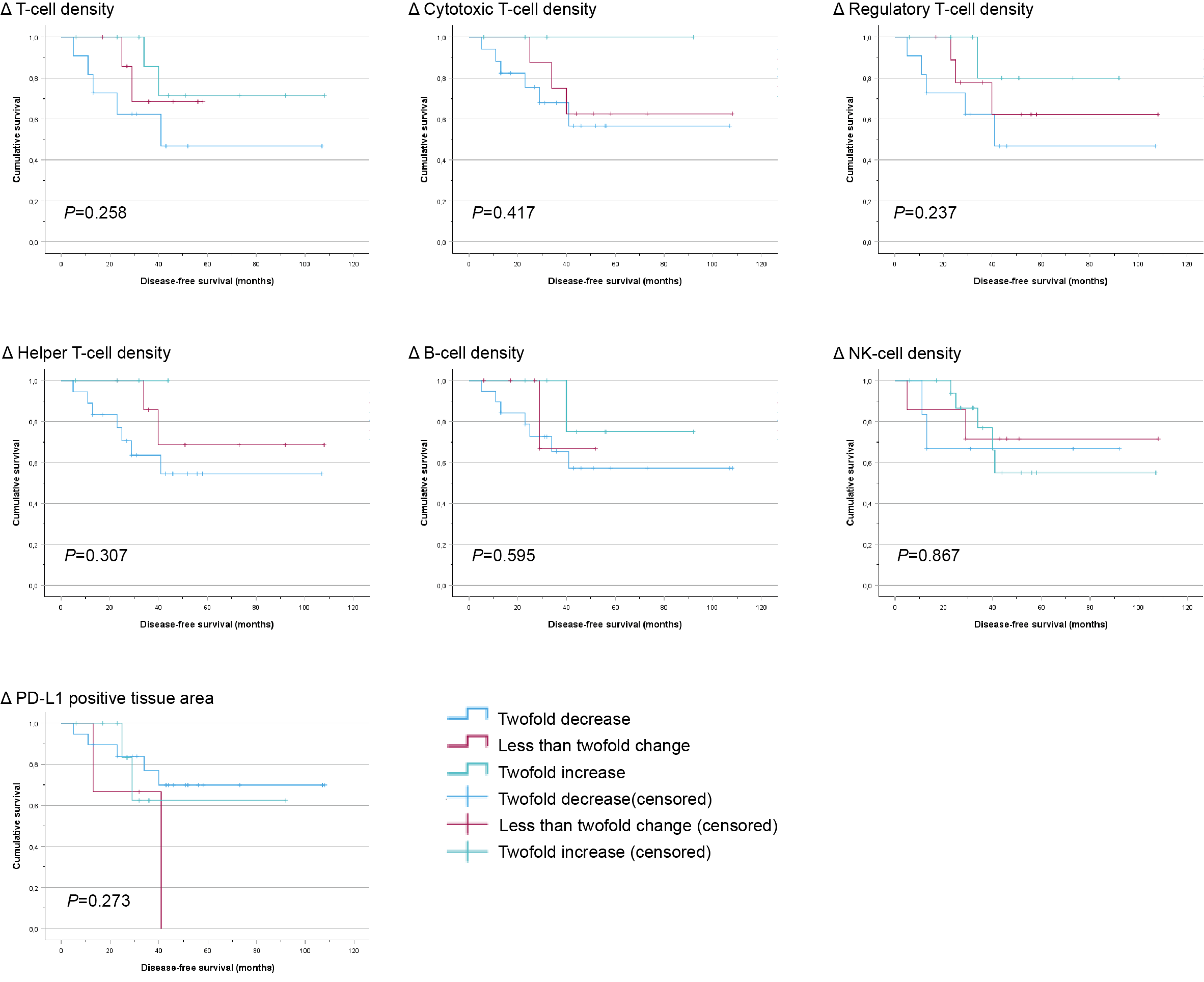
***
